# MULTIMODEL APPROACHES IDENTIFY CONSERVED ROLE OF TRP CATABOLIC PATHWAY IN THE REGULATION OF LIFE- AND HEALTHSPAN

**DOI:** 10.1093/geroni/igae098.2630

**Published:** 2024-12-31

**Authors:** Naci Oz, Alaattin Kaya

**Affiliations:** Virginia Commonwealth University, Richmond, Virginia, United States; Virginia Commonwealth University, Richmond, Virginia, United States

## Abstract

The activity of the conserved Kynurenine (KYN) pathway, a major catabolic pathway of Tryptophan (trp), is controlled mainly by the indoleamine 2,3‐dioxygenases IDO1, and IDO2, which catalyze the first rate-limiting step of trp catabolism by converting trp into kynurenic acid. The KYN pathway is the sole de novo NAD+ biosynthetic pathway, generating NAD+ from ingested trp. In addition, increased Ido1/2 activity, and intermediate metabolites such as quinolinic acid, as a result of normal aging, are mechanistically linked to inflammaging and neurodegenerative disorders such as Alzheimer’s diseases. Therefore, circulating kynurenines were suggested as a potential biomarker of frailty in humans. In contrast, decreased trp degradation has been beneficially implicated as a potent regulator of age-related diseases and lifespan. Although the KYN pathway is a viable therapeutic target against aging and age-related diseases, there have been no comprehensive studies in mice to understand the role of the KYN pathway in regulating life- and healthspan. To understand the conserved effect of the KYN pathway in modulating life- and healthspan, we developed individual and combined whole-body knockout transgenic mouse strains lacking genes IDO1, IDO2 or IDO1/IDO2. Using multi-omics approaches, we comprehensively characterized the tissue and sex-specific biological age and molecular and metabolic alterations mediated by KYN pathway deficiency. Along with the healthspan measurements, we showed a potentially conserved role that decreased trp catabolism might be a conserved modulator of life- and healthspan.

